# GaAs_1-*x*_Bi_*x*_ growth on Ge: anti-phase domains, ordering, and exciton localization

**DOI:** 10.1038/s41598-020-58812-y

**Published:** 2020-02-06

**Authors:** Tadas Paulauskas, Vaidas Pačebutas, Andrejus Geižutis, Sandra Stanionytė, Evelina Dudutienė, Martynas Skapas, Arnas Naujokaitis, Viktorija Strazdienė, Bronislovas Čechavičius, Mária Čaplovičová, Viliam Vretenár, Rafał Jakieła, Arūnas Krotkus

**Affiliations:** 1 Center for Physical Sciences and Technology, Saulėtekio al. 3, Vilnius, 10257 Lithuania; 2 STU Centre for Nanodiagnostics, University Science Park Bratislava Centre, Slovak University of Technology, Vazovova 5, Bratislava, 811 07 Slovakia; 3 Polish Academy of Sciences, Institute of Physics, Laboratory of X-ray and Electron Microscopy, al. Lotników 32/46, Warsaw, 02-668 Poland

**Keywords:** Materials science, Nanoscience and technology

## Abstract

The dilute bismide alloy GaAs_1-*x*_Bi_*x*_ has drawn significant attention from researchers interested in its fundamental properties and the potential for infrared optoelectronics applications. To extend the study of bismides, molecular-beam heteroepitaxy of nominally 1.0 eV bandgap bismide on Ge substrates is comprehensively investigated. Analysis of atomic-resolution anti-phase domain (APD) images in the direct-epitaxy revealed a high-density of Ga vacancies and a reduced Bi content at their boundaries. This likely played a key role in the preferential dissolution of Bi atoms from the APD interiors and Bi spiking in Ge during thermal annealing. Introduction of GaAs buffer on offcut Ge largely suppressed the formation of APDs, producing high-quality bismide with single-variant CuPt_B_-type ordered domains as large as 200 nm. Atomic-resolution X-ray imaging showed that 2-dimensional Bi-rich (111) planes contain up to *x* = 9% Bi. The anomalously early onset of localization found in the temperature-dependent photoluminescence suggests enhanced interactions among Bi states, as compared to non-ordered samples. Growth of large-domain single-variant ordered GaAs_1-*x*_Bi_*x*_ films provides new prospects for detailed analysis of the structural modulation effects and may allow to further tailor properties of this alloy for optoelectronic applications.

## Introduction

The development of dilute Bi-containing alloy GaAs_1-*x*_Bi_*x*_, referred to here as bismide, in recent years has been driven by its potential to extend III-V semiconductor devices into the infrared range. Incorporation of Bi into GaAs matrix leads to a large narrowing of the bandgap, up to ~90 meV/%, as compared to, e.g., by alloying with In (~10 meV/%)^1,2^. The bismide also has strong spin-orbit coupling effects on the electronic band structure, which is unique among III-V alloys. GaAs_1-*x*_Bi_*x*_ with concentrations *x* > 10% produces a separation of the split-off valence band energy that exceeds the bandgap, leading to suppression of the Auger recombination channel involving this band^3,4^. The bismide can be grown on GaAs substrates and is a promising candidate for several applications, including infrared lasers, heterojunction bipolar transistors, spintronics, and multi-junction solar cells^1,5,6^.

GaAs_1-*x*_Bi_*x*_ shares many similarities with GaAs_1-*x*_N_*x*_, which also produces a large bowing of the GaAs bandgap. Both bismide and the nitride show dual behavior characteristics – that of an alloy as well as of a highly-doped isoelectronic impurity material^7–9^. The bandgap reduction occurs due to the interaction of resonant Bi and N states in the proximity of GaAs valence and conduction band edges (VBE, CBE), respectively. While individual Bi and N atoms produce bandgap bowing and potential fluctuations, stochastically incorporated pairs (Bi-Bi, N-N) or clusters are thought to be a source of the local density of states that lead to the pronounced charge carrier localization and scattering in these alloys^10–13^. The bismide has presently emerged as a more promising candidate for photovoltaics (PV) since the trap states are formed near its VBE, whereas they occur near CBE in the nitride. High electron mobilities and lifetimes can thus be preserved in the former^14–17^. A lattice-matched 1.0 eV bandgap absorber material is highly desirable for III-V multi-junction (M-J) PV applications. The bandgap combination of the industry-standard 3-J device GaInP(1.9)/GaAs(1.4)/Ge(0.7) is non-ideal for space or terrestrial concentrated-sun conditions. Nearly 5% efficiency points could be added by replacing the Ge bottom cell with a 1.0 eV material, and as much as 10% by incorporating it into 4-J cells atop Ge^18,19^. The high absorption coefficient of 1.0 eV GaAsBi and less than 0.05% lattice-mismatch to GaAs has granted this material a thorough study within the PV community^20,21^.

Incorporation of Bi into III-V compounds requires low growth temperatures, typically below 400 °C, and a near-stoichiometric flux ratio of III/V elements due to the alloy miscibility gap^1,22,23^. There is a relatively small window of growth parameters that allow for high-quality bismides free of Bi and Ga droplets on the surface. Deviations from the growth conditions also lead to phase-separated domains and spontaneous nanostructure formations^24–26^. Furthermore, many recent studies challenge the assumption that Bi is incorporated randomly. Instead, CuPt-type ordering of group-V elements is often observed near the optimal GaAsBi growth conditions, an effect that is driven by surface reconstruction dynamics^27–30^. Spontaneous CuPt-type ordering in III-V semiconductor alloys typically occurs along two of the four <111> body diagonals, primarily along two <111> B, which is termed CuPt_B_^31^. Besides, the use of vicinal substrates favors the formation of a single CuPt_B_ subvariant. The ordering reduces zincblende cubic F$\overline{43}$m symmetry to trigonal R3m and can lead to several effects, including bandgap narrowing, valence band splitting, the polarization of light, and anisotropic strain^31–34^. No such ordering-induced effects have yet been reported in GaAsBi alloys.

An attendant challenge for the bismide-based PV development is its growth on Ge substrates, as one is faced with the epitaxy of a polar material on a non-polar substrate. Mixed nucleation of group-III and V elements and random surface steps on the substrate can produce anti-phase domains (APD), which degrade device performance^35–37^. As of this writing, the attempt to grow GaAs_1-*x*_Bi_*x*_ on Ge substrates, to the best of our knowledge, was made only once^38^. The authors synthesized bismides with up to 5% Bi on offcut (001) Ge substrates using Ga initiated migration-enhanced molecular-beam epitaxy (MEE-MBE). Employing a low-temperature MEE-MBE buffer in the first stage of growth, combined with a Ge substrate offcut, is a common practice to eliminate APDs in the GaAs-Ge heteroepitaxy^35,39,40^. Numerous triangular APDs in the first-few tens of nanometers were nevertheless found in the bismide, but an otherwise APD-pit-free surface, as well as a suppressed Ge diffusion into the film.

To extend the study of this heteroepitaxy, bulk bismide samples with nominally 1.0 eV bandgap are grown by MBE on vicinal Ge substrates and investigated using aberration-corrected scanning transmission electron microscopy (STEM) and atom-counting-based numerical techniques, X-ray diffraction and reciprocal-space mappings (XRD-RSM), temperature-dependent photoluminescence (PL), and secondary ion mass spectroscopy (SIMS). Rapid-thermal annealing (RTA) studies are also performed.

## Results and Discussion

### Direct epitaxy of GaAsBi on offcut Ge substrates

**Figure 1:**
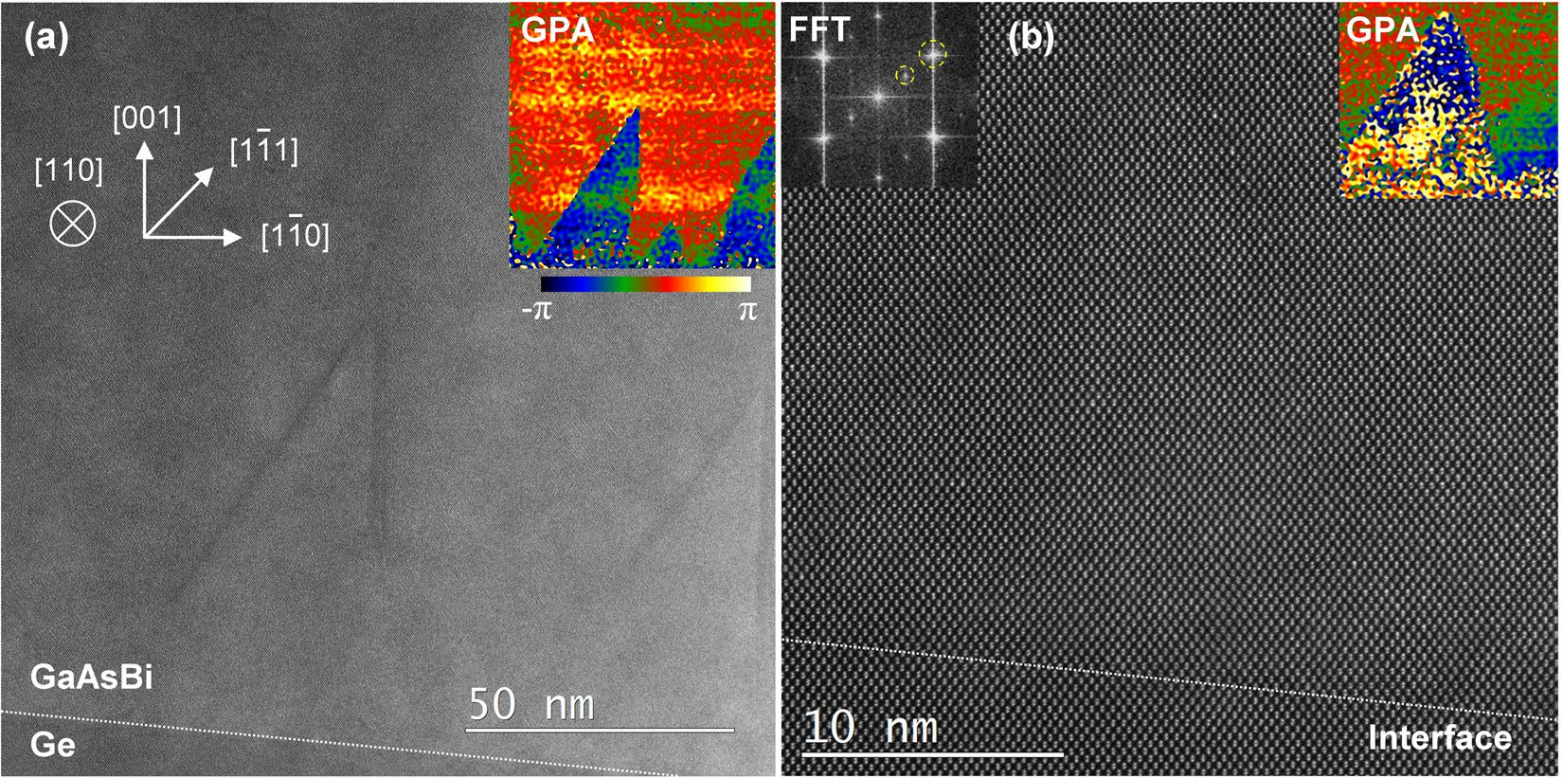
(**a**) A low-magnification HAADF image of the bismide sample S1 near the interface with Ge. A pair of asymmetric APDs are visible. Indicated crystallographic orientations are common for (**a**,**b**). The geometrical phase-map inset clearly outlines the APDs. (**b**) Atomic-resolution HAADF image near the interface showing an APD in the center, which is also outlined in the GPA inset. Small patches of single-variant CuPt ordering is seen in the lower right corner and confirmed in the Fourier transform (FFT) of the image.

The first sample studied here is an epitaxial bismide film synthesized directly on an offcut Ge (001) substrate (as-grown sample S1, see Sample synthesis in Methods). Structural characterization using STEM high-angle annular dark-field (HAADF) imaging was performed to analyze the sample, which was prepared such that the offcut [1$\bar{1}$0] direction is orthogonal to the HAADF imaging axis [110], allowing for the interface to be viewed edge-on. The scanning direction in STEM is aligned to set the [001] direction parallel to the vertical direction of the page so that the interface is seen tilted with respect to the horizontal in the amount of the offcut angle. Low-magnification HAADF image in Fig. 1(a) shows typical lattice features, indicating a high-density of APDs in this sample. A more systematic study is needed for the direct epitaxy in regards to the elimination of APD defects in GaAsBi. We note that the direct epitaxy could be bypassed in the M-J photovoltaic applications by first growing a highly-doped GaAs layer, or other III-V materials with established deposition recipes, which would act as the front-surface-field before a tunnel-junction interconnect. The analysis of a bismide sample grown over a GaAs buffer layer (sample S2) will be presented in the following section. We now turn to investigate the structure of APDs generated in the direct epitaxy on Ge.

The HAADF contrast in Fig. 1(a) is dominated by large APDs with a distinctive asymmetric triangular shape. The color-coded inset shows a geometrical phase analysis (GPA) phase map of the STEM image employing [002]* reciprocal lattice vector^41^. It highlights the APDs as the phase changes by π crossing into their interior. The asymmetric APDs that have boundaries with outward normal vectors [1$\bar{1}$0]//[$\bar{1}$11] are common in this bismide. The darker strips in HAADF images at the APD boundaries suggests a reduced Bi content. The boundaries of these domains do not seem to reach the interface, but rather intersect smaller APDs near the interface. Figure 1(b) shows an atomic-resolution HAADF image of an APD at the interface. The (1$\bar{1}$0) boundary here seems to have kinks, and shifts to a (1 $\bar{1}\,$1) plane near the apex before intersecting the ($\bar{1}$11) APD boundary. Small 2–5 nm domains of single subvariant CuPt_B_-type ordered bismide on (1$\bar{1}$1) planes can be seen outside the APD. A Fourier transform of the image is shown in the inset with the marked superlattice 1/2[1$\bar{1}$1]* Bragg spot next to [1$\bar{1}$1]*, indicating the presence of ordered domains.

### STEM image analysis of GaAsBi anti-phase domains

**Figure 2:**
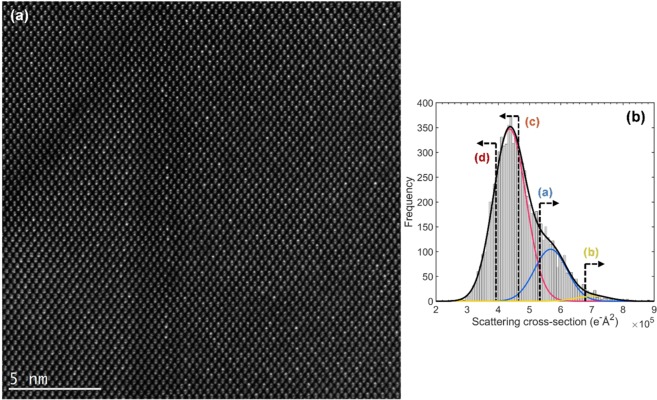
(**a**) Atomic-resolution HAADF image of a typical asymmetric triangular APD near its vertex. For crystallographic directions refer to Fig. 1(a). Note the reversal of atomic-dumbbell polarities in the interior of the APD. (**b**) A histogram of atomic-column scattering cross-sections (SCS) in Fig. 2 (**a**). The SCS distribution is tentatively fitted with three Gaussians. Vertical dashed lines with arrows refer to various SCS distributions mapped in Fig. 3.

Atomic-resolution HAADF image of a representative asymmetric triangular APD close to its vertex in sample S1 is shown in Fig. 2. GaAs crystal viewed along a <110> -type direction appears as a collection of closely-spaced Ga and As atomic-columns, so-called “dumbbells”. Pure Ga and As columns cannot be easily told apart since the HAADF contrast scales with an atomic-number as ~Z^2^, (Ga Z = 31, As-33, Bi-83). A large inner-collection angle of the HAADF detector was chosen here to enhance the contrast of Bi-containing columns^42,43^. Since Bi mainly replaces As atoms, this allows for distinguishing of group-V columns. The APD position and its boundaries in Fig. 2(a) can be clearly seen by noting the position of Bi atoms within [001] oriented dumbbells. In particular, Bi rich columns are in the lower half of each dumbbell within the APD, and this polarity switches outside the APD. Note that (1$\bar{1}$0) APD boundaries contain both, group III-III (Ga-Ga) and V-V (such as As-As, As-Bi, Bi-Bi) element wrong-bonds in equal numbers, whereas ($\bar{1}$11) boundaries have only Ga-Ga wrong bonds. The polar variant of the {111} boundary having group V-V wrong bonds was not observed in this sample, which is probably related to the dominant bismide (001) growth surface polarity. Theoretical studies of GaAs APDs suggest that {110}-type boundaries have the lowest formation energy due to their stoichiometry and charge transfer among Ga-Ga and As-As wrong bonds^44^. However, inclined planes, such as {111}, {112}, or {113}, can facilitate the annihilation of anti-phase boundaries and may lower the total systems’ energy. More studies are needed to elucidate whether the offcut or other growth factors favour the (1$\bar{1}$0)//($\bar{1}$11) boundaries in this bismide. Point defects can also alter the anti-phase boundary formation energies, and their distributions are investigated next.

**Figure 3:**
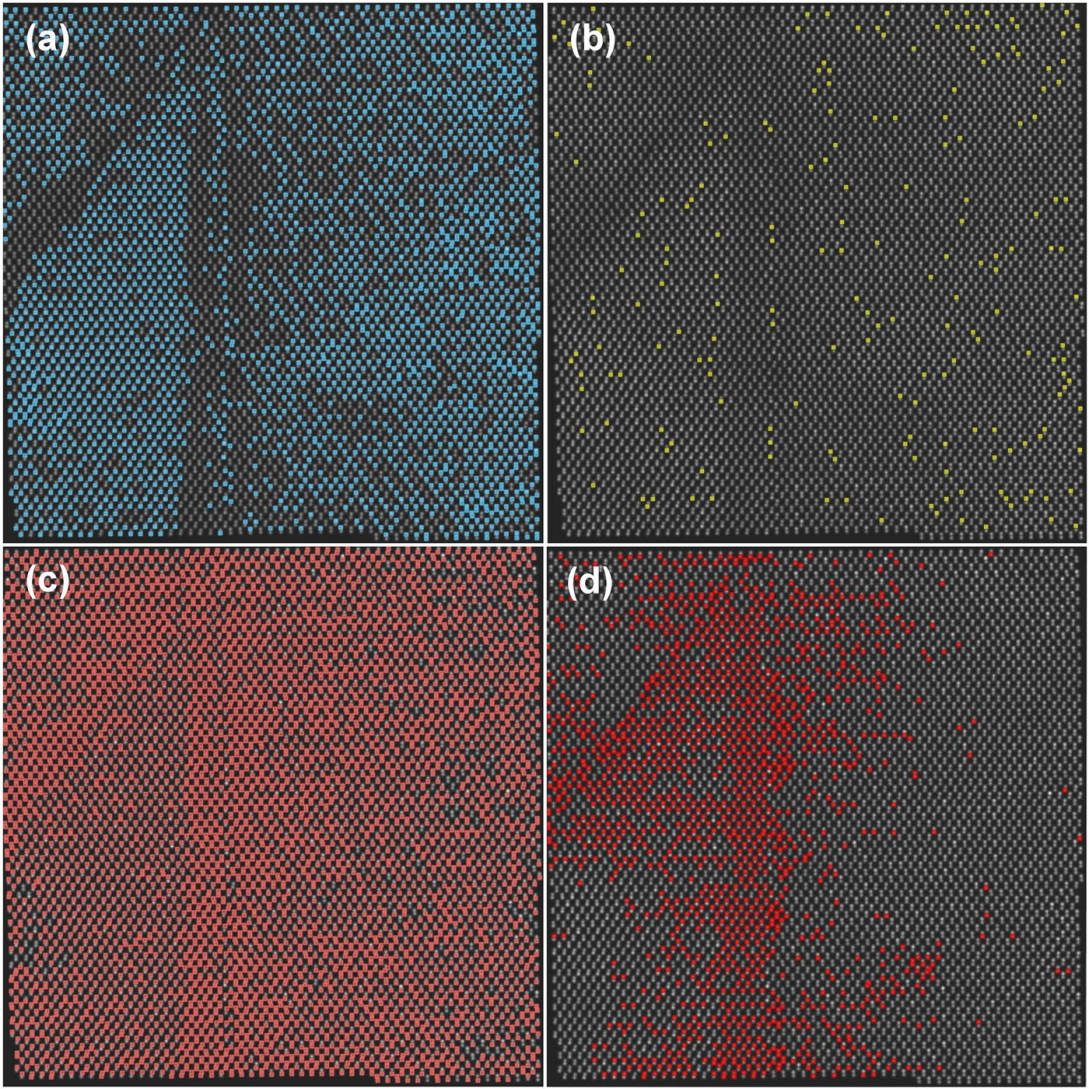
SCS mappings of the HAADF image in Fig. 2(a), with different SCS regions indicated in the histogram in Fig. 2(b). (**a**) Distribution of atomic-columns with SCS that are greater than (>) 5.4 e^−^Å^2^, which selects Bi-containing columns. (**b**) The distribution of SCS > 6.8 e^−^Å^2^, indicating columns with the highest Bi-content. (**c**) SCS < 4.7 e^−^Å^2^, which mainly selects Ga columns, and also the weaker scattering group-V columns where outlined. (**d**) SCS < 3.9 e^−^Å^2^, shows the distribution of Ga columns with low SCS values, and very weakly scattering group-V columns.

Numerical quantification of the entire HAADF image in Fig. 2(a) was performed to gain further insights. The StatSTEM algorithm works by finding individual atomic-columns and fits each column with a Gaussian^45^. A new model image is thereby produced composed of a superposition of Gaussian peaks. The dynamical scattering cross-section (SCS) of an atomic-column -*i* is defined by integrating the volume under the *i*^th^ Gaussian peak. The key factors determining the magnitude of a SCS are the type of atoms and their numbers in the column (see Methods for more details). The distribution of scattering cross-sections in Fig. 2(b) shows the count of atomic-columns in a given SCS range. Dashed vertical lines with arrows refer to various SCS regions, as mapped in Fig. 3 and discussed shortly. The histogram is tentatively fitted with three Gaussians, whose meaning can be interpreted as follows. Firstly, pure Ga and As columns, due to their similar Z-number, produce a strongly overlapping distribution of SCS, which gives rise to the tallest single Gaussian (red) centered at ~4.4 e^−^Å^2^. Plotting the spatial distribution of the lower end of the Gaussian predominantly picks Ga atomic-columns, as can be inspected from the dumbbell polarity. A fraction of As atoms are replaced by Bi, and so the SCS distribution of otherwise pure As columns is now extended with the extra Gaussian shoulders since Bi atoms scatter electrons much stronger to the high-angle annular detector. Atomic-column configurations with a different number of Bi atoms in them produce broadening of the SCS, which are tentatively represented here by two Gaussians (blue, yellow). We note that to establish SCS values of atomic-column configurations with various Bi distributions, numerical image simulations are needed to build a library for fitting to normalized experimental HAADF images. This, however, is beyond the scope of the present study.

Figure 3 shows the spatial distribution of scattering cross-sections in Fig. 2(a), plotted on the model structure by outlining the atomic-columns with colored squares. In Fig. 3(a), the distribution of atomic-columns with SCS > 5.4 e^−^Å^2^ is plotted, which corresponds to the position in Fig. 2(b) marked with a dashed vertical line where the Gaussian associated to columns with Bi atoms in them overtakes in frequency the Gaussian associated with pure Ga and As columns. This distribution indicates that (1$\bar{1}$0) APD boundary plane contains Bi atoms, but its concentration is diminished immediately on either side of it, forming thin 1–3 nm Bi-deficient regions. The ($\bar{1}$11) boundary region also shows reduced Bi content. The APD exterior exhibits small patches of CuPt-ordered domains, whereas no ordering is found in the APD interior in this projection. The outlined squares on group-III atomic-columns in Fig. 3(a) distribution indicate there a likelihood of hetero-antisite defects Bi_Ga_. Figure 3(b) shows SCS > 6.8 e^−^Å^2^, and is indicative of columns that have the largest Bi content in them. Figure 3(c) shows spatial distributions of SCS < 4.7 e^−^Å^2^, which mainly picks all group-III columns, and also group-V columns with lower SCS due to, e.g., vacancies or Ga_As_ antisites in them. An increased likelihood of such point defects on group-V columns appears to be concentrated near the APD boundaries, which is signified by the numerous outlined group-V columns. A similar distribution mapping is performed on the group-III columns by plotting SCS < 3.9 e^−^Å^2^ in Fig. 3(d), which targets the weakest scattering Ga-columns. The likelihood of Ga vacancies is concentrated at the APD apex and near its boundaries, clearly pronouncing the APD region. We note that no significant in-plane lattice strain was found at the APD (based on GPA and StatSTEM, not shown here), which could otherwise impact the image intensities and quantification of the SCS.

### Thermal annealing effects in the direct epitaxy

The effects of GaAs_1-*x*_Bi_*x*_ thermal annealing grown on GaAs substrates were investigated by several groups^46,47^. Even though the general trends were identified, the results are still ambivalent due to a spread in growth conditions. Firstly, the annealing is thought to reduce point defects generated in a low-temperature-grown GaAs lattice. The GaAsBi alloy stability at temperatures above ~600 °C and the observed onset of Bi segregation is dependent on the Bi fraction in samples, with higher Bi% typically seen as less stable, and also on the lattice quality before the annealing.

**Figure 4:**
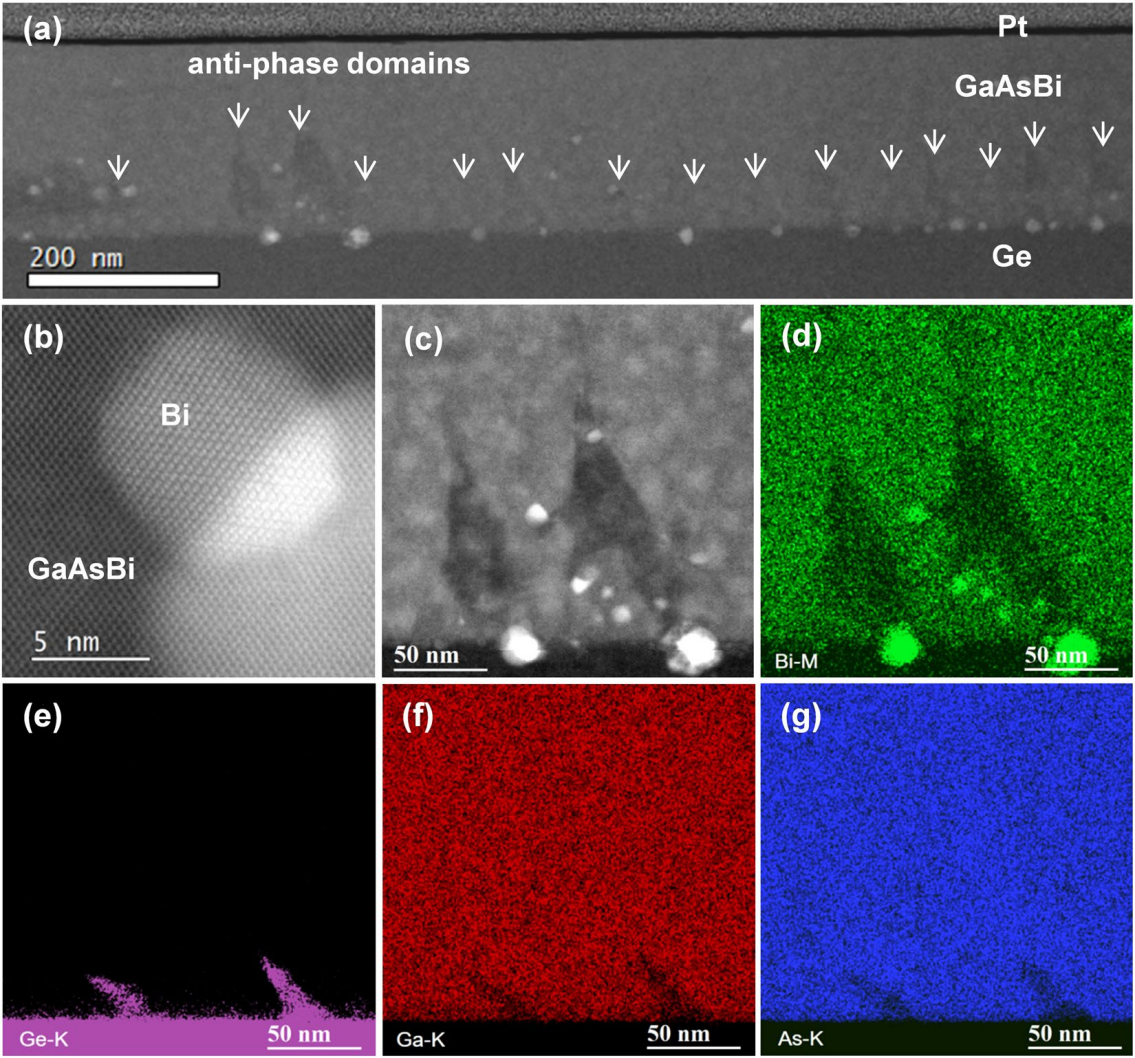
(**a**) HAADF image of sample S1 annealed at 600 °C. Anti-phase domains are indicated with arrows. (**b**) HAADF image of a rhombohedral Bi droplet formed within the bismide film. (**c**–**g**) Simultaneously acquired HAADF image, (**c**), and the EDS elemental mapping of Bi, Ge, Ga, and As (**d**–**g**), showing a pair of APDs near the interface with Ge.

In this section, STEM-EDS is employed to examine the crystal structure and chemical changes of the bismide sample S1 annealed at 600 °C in a rapid-thermal annealing (RTA) oven (see Methods). This temperature treatment is presented here since noticeable photoluminescence changes already occur at 600 °C, as compared to the as-grown S1 sample. A low-magnification HAADF image of the bismide is shown in Fig. 4(a). Numerous asymmetric triangular APDs can be seen (indicated with arrows) with a similar density as in the as-grown S1 sample, suggesting that the APD density did not change significantly. However, the APD interiors appear darker in the Z-contrast HAADF image, indicating a lower Bi concentration. Furthermore, Bi precipitates are found near all these Bi deficient domains. Bi particles were typically reported to assume a rhombohedral crystal structure within the GaAsBi matrix, and we indeed found numerous coherently aligned Bi crystallites, as illustrated in Fig. 4(b)^48^. Quite a few laterally elongated phase-separated domains were found in the annealed sample. Phase-separation can occur during growth, but can also be initiated and enhanced through a dissolution of Bi during a thermal treatment. The latter appears to be the case here since phase-separated domains are accompanied by Bi precipitates, which are virtually absent in the as-grown film. Interestingly, the APD interiors are found to consistently reject Bi, which then preferentially accumulates in droplets near the interface with Ge substrate. Some of the Bi particles clearly penetrate the Ge substrate. X-ray energy dispersive chemical mapping of the sample in Fig. 4(c–g) shows Bi precipitates and confirms that large portions of the APD interior, mainly upper parts towards their vertices, are Bi deficient. Ge out-diffusion from the substrate is also noticeable in the EDS images, accompanied by reduced Ga and As concentrations. Due to large size of Bi atoms, vacancy-mediated hopping of Bi atoms has been suggested as the primary means of Bi diffusion and clustering^16^. The higher density of Ga and As vacancies at the anti-phase domains, as presented in the previous section, could explain the preferential Bi dissolution from the APD interiors during high-temperature annealing. On the other hand, Ge diffusion along vacancies in GaAsBi APDs and a subsequent filling of Ge vacancies with Bi could be one way to interpret the spiking of Bi within the substrate.

### Single-variant CuPt-type ordering of bismide grown with GaAs buffer layer

**Figure 5:**
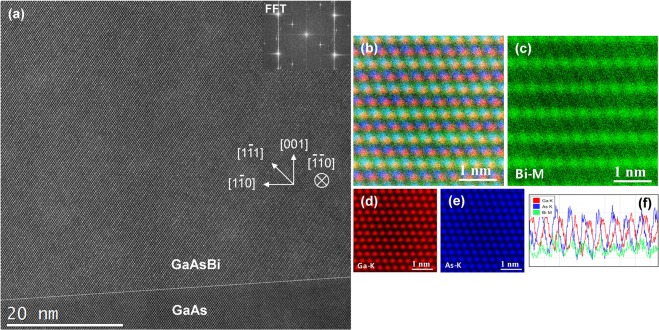
(**a**) HAADF image of single-variant CuPt-ordered bismide sample S2 near the interface with GaAs buffer layer. Crystallographic orientations and Fourier transform of the image are shown. (**b**–**e**) Atomic-resolution EDS images of the ordered bismide, with individual maps showing Bi, Ga, and As X-ray emissions, and their combined colour-overlaid map in (**b**). (**f**) Horizontally summed signal line-profile across an ordered Bi-rich (111) plane. Note that STEM scanning direction is set in the EDS to align ordered planes horizontally.

The as-grown sample S2 is analyzed next, which is composed of 230 nm thick nominally 1.0 eV GaAsBi deposited over 300 nm thick GaAs buffer layer on offcut Ge. The GaAs buffer was grown in three steps to reduce the density of APDs in GaAs and to minimize elemental interdiffusion across Ge-GaAs interface (see Methods)^39,40^. Secondary ion mass spectroscopy showed no significant diffusion of Ge into the GaAs buffer (Supplementary Fig. S1). Most of the APDs that were nevertheless generated in GaAs were buried within the buffer, leaving the bismide film virtually free of APDs. Figure 5(a) shows a representative HAADF image of the film with a GaAs buffer layer. STEM imaging direction in sample S2 is [$\bar{1}\bar{1}0$], and [001] is aligned vertically so that the GaAs-GaAsBi interface is inclined with respect to the horizontal. The preservation of the offcut angle and elimination of APDs had promoted the bismide growth that is uniformly single-variant CuPt_B_-ordered. The ordered domain sizes are at least ~230 nm (thickness of the film) with few small randomly interspersed areas showing a lower degree of ordering. As of this writing, this constitutes the largest CuPt-type domains stabilized in GaAsBi.

Atomic-column resolved EDS mapping of the bismide is presented in Fig. 5(b–f). The scanning direction in STEM was set to align the ordered (1$\bar{1}$1) planes horizontally. The ordering of Bi atoms is clear from Bi-*M* X-ray emissions in Fig. 5(c), and is also illustrated in Fig. 5(b), which is composed by overlaying EDS of all elements on the simultaneously acquired HAADF image. Line profiles across an ordered plane, shown in Fig. 5(f), indicate that Bi positions overlap As, confirming Bi_As_ substitution. By quantifying EDS signals along the ordered Bi-rich planes, we find that Bi concentration in them reaches *x*′ = 9% (+/−0.5%). The XRD-RSM measurements, presented in the following section, indicate Bi concentration in the lattice is *x* = 5.8% Bi, while the EDS results show *x* = 5.5%. In general, an ordered bismide GaAs_1-*x*_Bi_*x*_ is composed of alternating Bi-rich GaAs_1-(*x*+*η/2)*_Bi_*x*+*η/2*_ and As-rich GaAs_1-(*x*-*η/2)*_Bi_*x*-*η/2*_ monolayers along <111>, where *η* is the long-range order parameter^31^. Keeping that Bi concentration in the sample is *x* = 5.5%, while in the ordered Bi rich planes *x*′ = 9%, the order parameter is found *η* = *0*.*07*. Note that the *η*_*max*_ in a fully ordered GaAs_0.945_Bi_0.055_ would be *η* = *0*.*11*, while the maximal *η* = 1 is achieved in a fully ordered alloy at *x* = 50%.

### X-ray diffraction rocking-curve and reciprocal-space mapping

**Figure 6:**
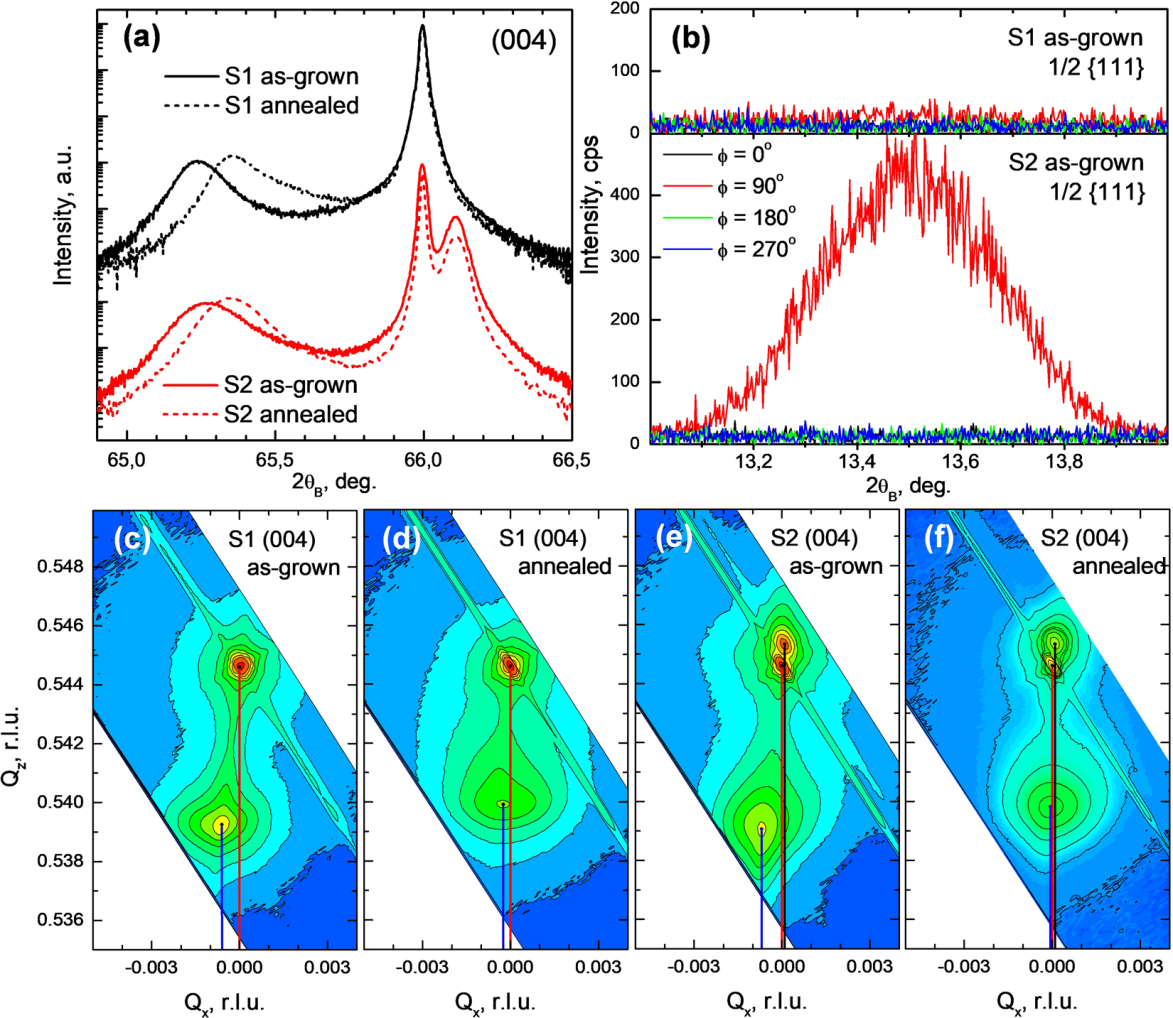
(**a**) XRD (004) reflection rocking-curves of the as-grown and annealed (600 °C) samples S1 and S2. (**b**) XRD measurement of CuPt-type ordering. The direct-epitaxy sample S1 shows little-to-none superlattice ½{111} reflections, while S2 clearly indicates ordering on a single set among four distinct sets of {111} planes. (**c**–**f**) Reciprocal-space mappings of (004) reflections of samples S1 and S2 before and after annealing (600 °C). The bismide layer tilt is visible in both as-grown samples, the angle decreases after the annealing.

Figure 6(a) shows X-ray diffraction (XRD) rocking-curve measurements of the bismide samples S1 and S2. Bi concentration in the as-grown samples S1 and S2 are *x* = 5.5% and *x* = 5.8%, respectively. The concentrations were computed using GaAs and fictitious GaBi lattice constants (see Methods), taking into account GaAsBi lattice relaxations obtained from the reciprocal-space maps (discussed below). Dashed lines in Fig. 6(a) also show XRD of the samples after annealing in RTA oven at 600 °C, indicating a decrease of Bi to 5.2% and 5.5% in S1 and S2, respectively. A decrease of Bi content in GaAsBi lattice is commonly observed in bismides after thermal annealing and can indicate Bi-segregation^46,48^. To investigate CuPt-type ordering, measurements of superlattice reflections were performed in skew-symmetric configurations to access all four distinct sets of {111} planes^49^. Only one set of planes show the superlattice reflections ½(111) in sample S2 in Fig. 6(b), which agrees with the STEM observations. This diffraction peak is almost absent in sample S1 grown directly on Ge (Fig. 6b, upper graph), suggesting that CuPt-ordered domains are small and scarce. The superlattice XRD peak intensity in sample S2 slightly decreases after thermal annealing (SI Fig. S2). Interestingly, a bismide sample grown directly on Ge substrate without an offcut showed weak 1/2{111} superlattice reflections on all four distinct sets (SI Fig. S2). This is likely due to mixed-polarity domains occurring in similar proportions.

RSM of (004) reflections of the as-grown and annealed samples S1 and S2 are presented in Fig. 6(c–f). Figure 6 (c,e) show that the as-grown samples S1 and S2 incurred a bismide layer tilt of 0.064° and 0.074°, respectively, with respect to Ge, indicating an additional leaning towards the offcut direction. This effect has been explained by Nagai, and arises here from the lattice-mismatch in the (001) direction between the epitaxial layer and Ge or GaAs, due to the offcut-introduced surface steps^34,50^. The epilayer tilt can be accommodated by a lattice strain without nucleation of dislocations if the lattice mismatch is not too large. Here, we find the layer tilt angles, *γ*, follow fairly closely the Nagai equation, $tan\gamma /tan\beta =({a}_{L}-{a}_{S})/{a}_{S}$, where *a*_*L*_ and *a*_*S*_ are the layer and substrate lattice constants (*a*_*z*_), respectively, and *β* is the substrate offcut angle^50,51^. Following the Nagai equation, the GaAs buffer layer is indeed tilted in the opposite direction by 0.001° due to its smaller lattice constant than Ge. After annealing at 600 °C, the tilt angles decrease, with final values 0.026° in S1, and 0.007° in S2. This indicates that dislocations having a Burgers vector with a tilt component opposite to the initial tilt direction nucleated during the lattice relaxation. Such decrease in tilt angles is observed in other zincblende materials, provided there are no sources for significant asymmetry in regards to nucleation and glide of dislocations on different sets of {111} planes^34^. RSM (004) reflections in Fig. 6(c,e) and also (115) (in SI Fig. S2), indicate that as-grown bismide layers are in tensile strain in the out-of-plane direction with lattice parameters (*a*_*z*_) 5.716 Å (sample S1) and 5.718 Å (sample S2). The in-plane lattice parameters (*a*_*x*_) are 5.657 Å (S1) and 5.660 Å (S2) (SI Fig. S2), which shows that sample S1 is fully compressively strained to the substrate, while ~9% of the S2 lattice is relaxed, probably due to their different thicknesses (100 nm S1, 230 nm S2). Annealing of both samples at 600 °C leads to further relaxation, with final lattice parameters (*a*_*x*_, *a*_*z*_) in S1 (5.669, 5.704) Å, and (5.666, 5.710) Å in S2, indicating the final relaxation is *R* = 37% in S1, and *R* = 25% in S2^52^.

### Photoluminescence properties

The photoluminescence properties of GaAsBi have been a subject of numerous studies^11,12,53–56^. Notable PL features of the bismide include a broad PL peak at room temperature, a red-blue-red shift of the temperature-dependent PL peak-energy position, integrated PL intensity plateau at intermediate temperatures, and the saturation of the Stokes-shift at elevated excitation powers. Present understanding asserts that two independent Gaussian-shaped local density-of-states (LDOS) components are required to explain the radiative properties. The first Gaussian distribution extends from the VBE and is attributed to the short-range disorder and fluctuations of Bi content. The second is offset by some 100 meV  from the VBE into the bandgap and is likely due to Bi-related complexes. Empirical rate-equations show that competition between excitons hopping among the LDOS and their thermalization to the mobility-edge with increasing temperature, whereby non-radiative channels become accessible, leads to the observed PL features.

**Figure 7:**
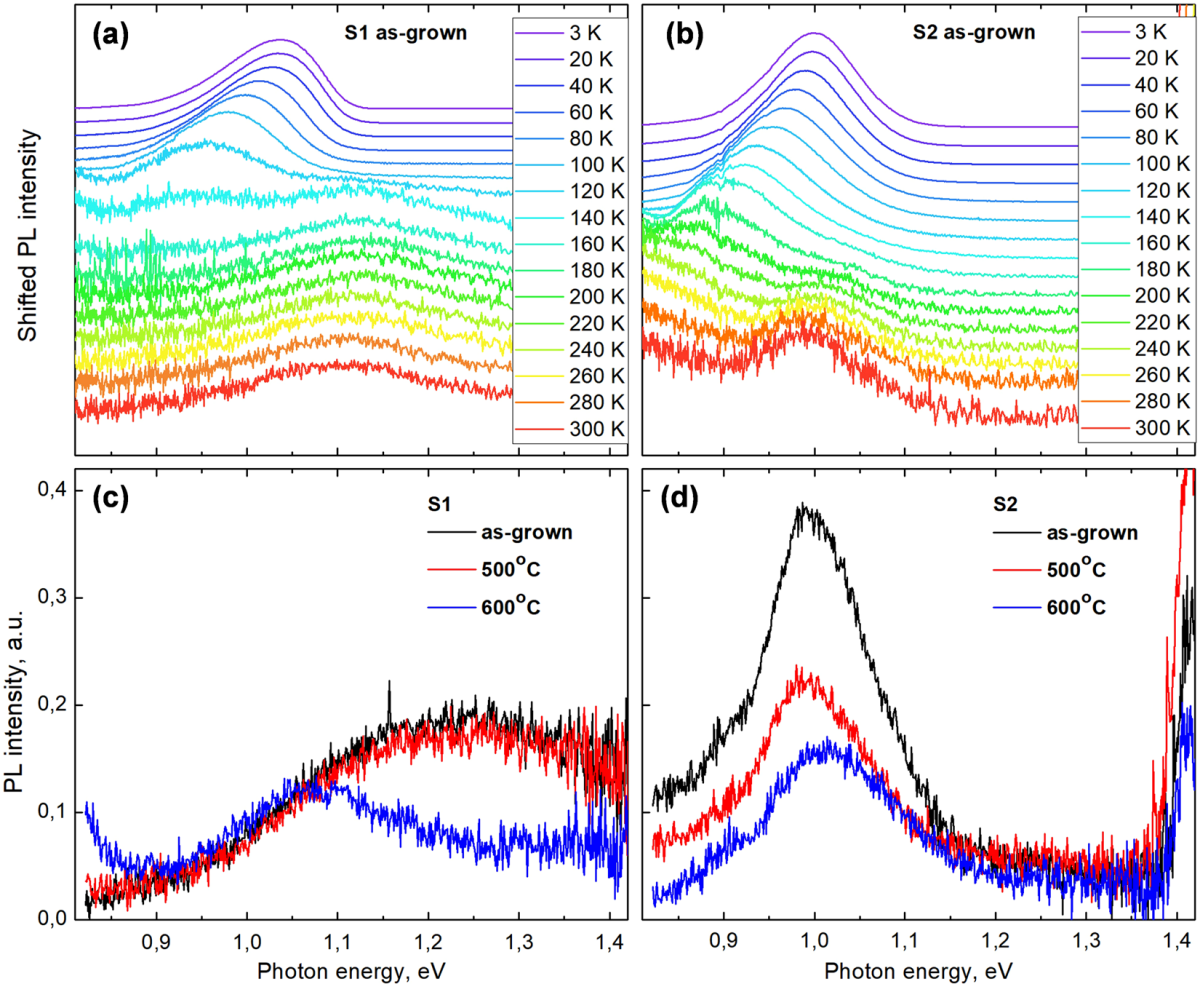
(**a**,**b**) Temperature-dependent photoluminescence of as-grown samples S1 and S2 in the temperature range 3–300 K. The spectra are normalized to the maximum of GaAsBi PL emission at each temperature and shifted vertically for clarity. (**c**,**d**) Room-temperature PL of the as-grown and annealed samples S1 and S2.

The temperature-dependent PL spectra of the as-grown sample S1 is shown in Fig. 7(a). It exhibits a very broad band-edge emission peak at 1.1 eV, likely due to the non-homogenous Bi content and a highly-defective lattice, as observed in STEM images. PL from the Ge substrate is also visible below ~0.8 eV. The temperature-dependent PL first shows a blue-shift due to the lattice contraction with decreasing temperature and is then followed by the characteristic red-blue shift. The onset of this effect is accompanied by the second peak, which emerges ~150 meV lower in energy, signifying trapping of excitons in deep states. As the temperature decreases still further, the excitons are unable to reach the deepest states and remain trapped at the shallower LDOS.

The temperature-dependent PL spectra of sample S2 in Fig. 7(b) shows narrower PL peaks and reflects a more uniform GaAsBi crystal, with band-edge emission at ~1.0 eV. Nevertheless, the emission from deep states seems strongly enhanced in this sample. Deep states start to dominate the PL intensity from temperatures 220–240 K, which is in contrast to sample S1 in Fig. 7(a), whereby the S-shift occurs at 140–150 K and is more typical for bismides. The anomalous onset of below-band-edge luminescence at relatively high-temperatures in S2 may reflect the pronounced CuPt-ordering. The ordering renders 2-dimensional Bi concentrations up to *x* = 9%, which is expected to produce stronger interactions among localized Bi states.

The room-temperature PL spectra of samples S1 and S2 annealed at 500 °C and 600 °C in an RTA oven are shown in Fig. 7(c,d). A somewhat different PL peak position of the as-grown sample S1, as compared to the S1 temperature-dependent PL peak at 300 K, could be due to Bi inhomogeneities across the sample. After annealing at 600 °C, sample S1 shows a small decrease in the band-edge emission peak intensity as well as the peak narrowing, and a red-shift to 1.07 eV. This emission energy adheres better to the expected bismide bandgap at *x* = 5.5% Bi concentration, as shown using XRD in the previous section, and suggests a beneficial rearrangement of the lattice produced by the RTA. Sample S2 grown over the GaAs buffer layer does not show any significant improvement in the PL after the annealing treatments. A decrease in the emission intensity could result from the partial lattice relaxation. The blue-shift visible after 600 °C RTA is likely due to the dissolution of Bi atoms from the lattice and is corroborated by the XRD-RSM measurements. This distinct response to annealing of samples S1 and S2 suggests that optimal annealing parameters of bismides need to be tailored based on their composition and as-grown lattice quality, such as defect content and Bi distribution modes.

### Summary and conclusions

Bulk ternary bismides GaAs_1-*x*_Bi_*x*_ with *x* = 5.5% and 5.8% were grown by MBE on offcut Ge substrates and analyzed in this study. The direct epitaxy on Ge lead to a high density of anti-phase domains with a characteristic asymmetric triangular shape, bounded by {111} and {110}-type crystal planes. Atomic-resolution analysis of APDs revealed that the dark contrast at their boundaries arises due to a reduced Bi content. The distribution of Ga vacancies was spatially mapped on STEM images employing computed scattering-cross sections, indicating a significant increase of these point defects at the APDs. This likely played a key role in the dissolution of Bi atoms from the interior of APDs and Bi spiking in Ge substrate during thermal annealing. Incorporation of a GaAs buffer layer was found to eliminate APDs in the bismide layer and a high-quality film was obtained. Moreover, single-variant CuPt-type ordered domains as large as 200 nm  were stabilized in the bismide. The anomalously early onset of strong localization in the temperature-dependent PL of the ordered sample suggests an underlying rearrangements of localized density of states and enhanced exciton coupling. These findings show that high-quality bismide can be grown on Ge substrates. However, a growth of subsequent layers atop the bismide can be complicated if it results in the bismides thermal annealing. Both sets of samples showed Bi seggregattion after annealing at 600 °C. The relationship of GaAs_1-*x*_Bi_*x*_ alloy stability to its initial lattice quality needs to be better understood. On the other hand, large-domain CuPt-type ordering encourages further studies and may provide another route to tailor bismides properties for optoelectronic device applications.

## Methods

### Sample synthesis

Bismide structures were grown by solid-source molecular-beam epitaxy (MBE) on *p*-type epi-ready (001) Ge substrates, supplied by the Axt, Inc., with 6° offcut towards an in-plane <110> -type direction. Two different samples were produced for this study: S1 consisting of a direct bismide epitaxy on Ge, while in S2, a GaAs buffer layer was grown first on Ge, and followed by a bismide film. The samples were each divided into three pieces, so that S1 and S2 sample sets each contains the as-grown, annealed at 500 °C, and annealed at 600 °C samples. MBE growth was performed in an SVT-A reactor equipped with metallic Ga and Bi sources, and a two-zone valved-cracker source using As_2_ dimers. The deoxidation and sample growth temperature was monitored by a thermocouple-based controller. To remove native oxides from the substrates and prepare the Ge surface for GaAs deposition, the wafers were heated at 640 °C for 15 min under arsenic flux. In samples with GaAs buffer layers, migration-enhanced epitaxy (MEE) was used at 350 °C to deposit 20 periods consisting of 20 gallium and 20 arsenic atomic layers and was followed by 50 nm of GaAs grown at 500 °C. The remaining ~250 nm of GaAs buffer was grown at 600 °C. This three-level scheme of GaAs growth was used to reduce elemental interdiffusion across the Ge/GaAs interface, and to minimize APD density in the buffer layer^35,39,40^. GaAsBi films were grown at 350 °C. Before the growth was initiated, the shutter was opened to give a bismuth cover over GaAs buffer or Ge wafer surfaces. The growth rate used in both samples was 400 nm/h. The BEP ratio of As_2_ to Ga was fixed in the range from 1.063 to 1.1, and Bi/Ga ratio was 0.35–0.37. Post-growth *ex-situ* annealing of the samples was done in a rapid-thermal annealing (RTA) oven UniTemp1300 under a nitrogen atmosphere. The sample surfaces were covered by a GaAs substrate to minimize arsenic loss. Annealing duration at the set temperature (500 °C or 600 °C) was kept to 180 seconds in all samples.

### STEM

Cross-sectional TEM samples were prepared by FEI Helios Nanolab 650 dual-beam microscope equipped with an Omniprobe manipulator using the lift-out technique. The samples were thinned and polished to ~30 nm thickness and argon-oxygen plasma-cleaned before loading into the microscope. STEM high-angle annular dark-field (HAADF) images were acquired using cold-field-emission double aberration-corrected JEOL JEM-ARM200CF operated at 200 kV. Inner-collection semi-angle of the HAADF detector was set to 90 mrad, the probe convergence semi-angle was 22 mrad. X-ray energy dispersive spectroscopy (EDS) mappings were acquired using 0.98 steradian solid-angle windowless silicon drift-detector JED-2300 (JEOL, Japan) mounted in the STEM instrument. For the atomic-resolution EDS, the probe current was set to 200 pA, 0.1–0.2 msec pixel dwell time. Wiener filtering and sample drift-correction were applied, 5–6 sweeps were accumulated for the atomic-resolution EDS images.

### Image analysis with StatSTEM

HAADF image analysis was carried out using StatSTEM v3.0 software suite for Matlab^45^. To reduce the number of search parameters and ensure algorithm stability, single-width 2D Gaussian functions were fitted to the atomic-column positions. In contrast to a commonly employed Voronoi cell segmentation, the current approach allows for more accurate quantification of HAADF images that contain closely-spaced atomic-columns, e.g. <110> GaAs (~1.4 Å). The values of SCS primarily depend on the number and size of atoms in the column. Columnar distortions and the related broadening of SCS distributions are expected due to large Bi atoms, although this effect is expected to be small compared to e.g., influence of point defects. The positions of Bi atoms along the column will also influence the HAADF intensity and the SCS, since the electron probe intensity decreases with increasing specimen depth. We assume that the sample thickness does not vary significantly (<1 unit cell) over the field-of-view. Furthermore, experimental instabilities, such as scanning “jitter”, sample drift, and STEM electron-source fluctuations add to the SCS broadening. The magnitude of these factors is not significant, as can be inspected in the experimental HAADF images, and they are of secondary importance in the presented semi-quantitative analysis.

### XRD-RSM

X-ray diffraction (XRD) rocking curves (2theta/omega scan) were measured by a Rigaku Smartlab (Japan) diffractometer with 9 kW rotating Cu anode X-ray generator. Ge(400) × 2 monochromator was employed to select Cu Kα1 line for the diffraction experiments. Diffraction patterns were registered with scintillation detector SC-70 (for 2D measurements, a linear Dtex/Ultra detector was employed). Epitaxial layer tilt with respect to the substrate was calculated from (004) RSM and accounted for in the (115) RSM analysis. Symmetric (004) and asymmetric (115) plane RSM were used to evaluate layer relaxations, lattice parameters, and Bi concentrations. For the analysis, the following lattice constants were used: GaAs = 5.653 Å, GaBi = 6.324 Å. Measurements of ½{111} superlattice reflections were performed in skew-symmetric configurations^49^.

### SIMS

The secondary ion mass spectrometry (SIMS) sample characterization was performed using CAMECA IMS6F system. Cesium (Cs+) primary beam was used at 14.5 keV energy and current kept at 55 nA. The size of the raster was about 150 × 150 micrometers, secondary ions were collected from the central region 60 micrometers in diameter. Secondary ions Ge, Bi, Ga, and As as reference signals, were collected. Ge and Bi concentrations were calculated based on RSF’s for GaAs, as measured with Cs beam. Bi concentration 1e21 at/cm^3^ correspond to 2,3% at. content of element (i.e. *x* = 4.6%).

### PL

Photoluminescence (PL) measurements were performed using a monochromator (Andor SR-500i, 500 mm focal length), and a thermoelectrically cooled InGaAs photodetector. For the excitation, diode-pumped solid-state laser emitting at 532 nm wavelength and intensity of ~100 W/cm^2^ were used. Samples were mounted in a closed-cycle helium cryostat with a temperature controller. Spectra were normalized at each temperature to the maximum of GaAsBi PL peak and shifted vertically for clarity.

## Supplementary information

Supplementary information.
