# Supplementary material for: GaAs1-xBix growth on Ge: anti-phase domains, ordering, and exciton localization
Source: Sci Rep. 2020 Feb 6;10:2002. doi: 10.1038/s41598-020-58812-y (PMC7005183; doi:10.1038/s41598-020-58812-y)

Supplementary Information

**GaAs_1-x_Bi_x_ growth on Ge: anti-phase domains, ordering, and exciton localization**

Tadas Paulauskas,^1*^ Vaidas Pačebutas, ^1^ Andrejus Geižutis,^1^ Sandra Stanionytė,^1^ Evelina Dudutienė,^1^ Martynas Skapas,^1^ Arnas Naujokaitis, ^1^ Viktorija Strazdienė,^1^ Bronislovas Čechavičius,^1^ Mária Čaplovičová,^2^ Viliam Vretenár,^2^ Rafał Jakieła,^3^ & Arūnas Krotkus^1^

^1^Center for Physical Sciences and Technology, Saulėtekio al. 3, Vilnius, Lithuania

^2^STU Centre for Nanodiagnostics, University Science Park Bratislava Centre, Slovak University of Technology, Vazovova 5, Bratislava, Slovakia

^3^Polish Academy of Sciences, Institute of Physics, Laboratory of X-ray and Electron Microscopy, Warsaw, Poland

*tadas.paulauskas@ftmc.lt

**Figure S1.** (a) Secondary ion mass spectroscopy (SIMS) of the as-grown sample S2. The diffusion of Ge into the bismide’s front surface is visible, likely due to the evaporation of Ge from the substrate’s back-side and its subsequent adsorption on the top surface. Slightly higher Bi concentration is seen on the top surface, which could be due to occasional Bi droplets found on the sample surface. GaAsBi layer is 200 nm thick. (b) A reference atomic-resolution HAADF image of the ordered as-grown bismide sample S2 showing single variant CuPt-type ordering.


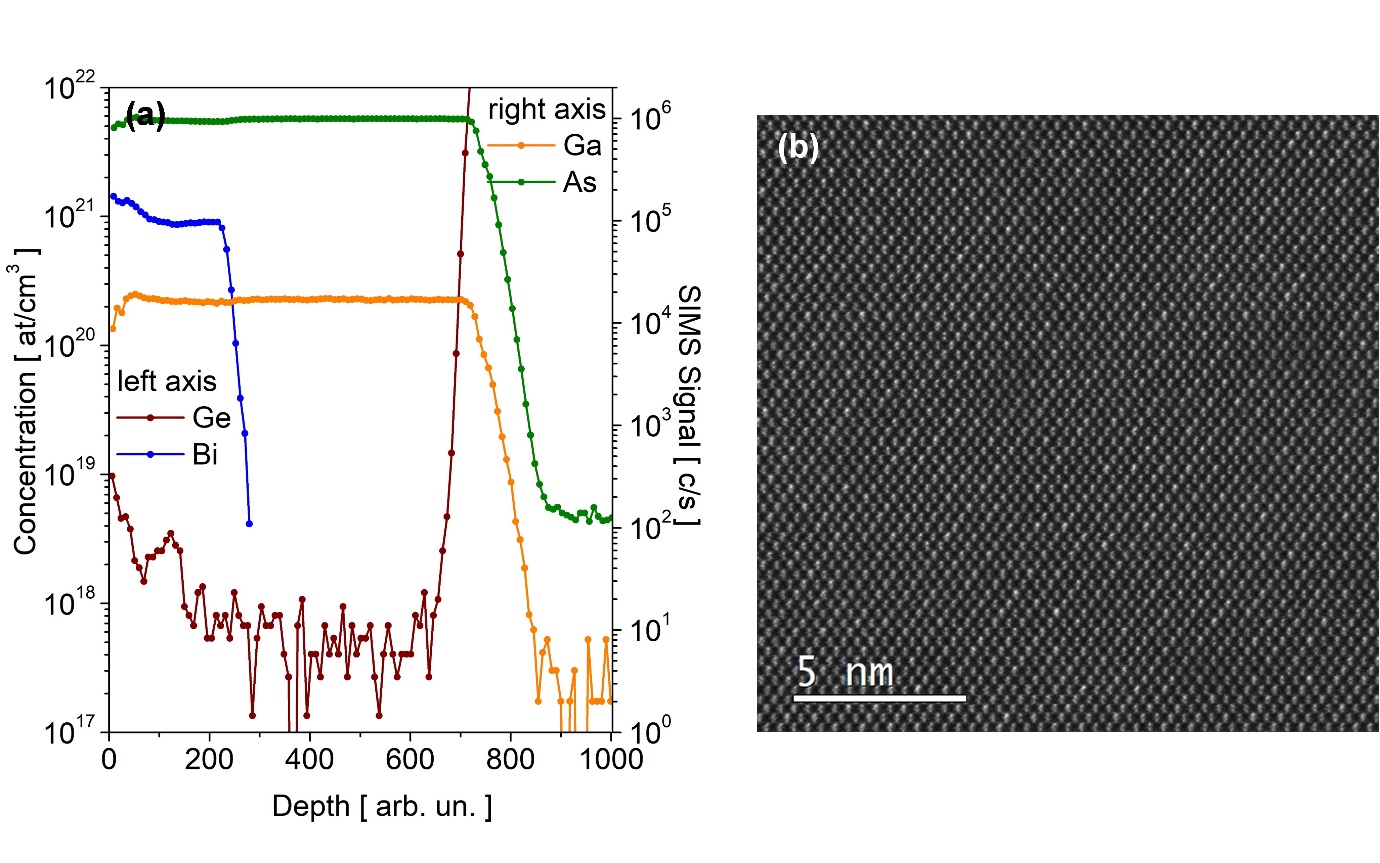


**Figure S2.** (a) XRD rocking-curves showing weak superlattice ½{111} reflections on all four distinct sets of {111} planes in the bismide sample grown directly on a flat (001) Ge substrate. (b) XRD rocking-curve of the S2 sample after annealing at 600°C, indicating that the single-variant CuPt-ordering slightly decreases, but still remains pronounced. (c-f) Reciprocal-space mappings of (115) reflections of bismide samples S1 and S2 before and after annealing at 600°C. The in-plane (Q_x_), and out-of-plane (Q_z_) lattice relaxations are computed and quoted in the main text based on the RSM.


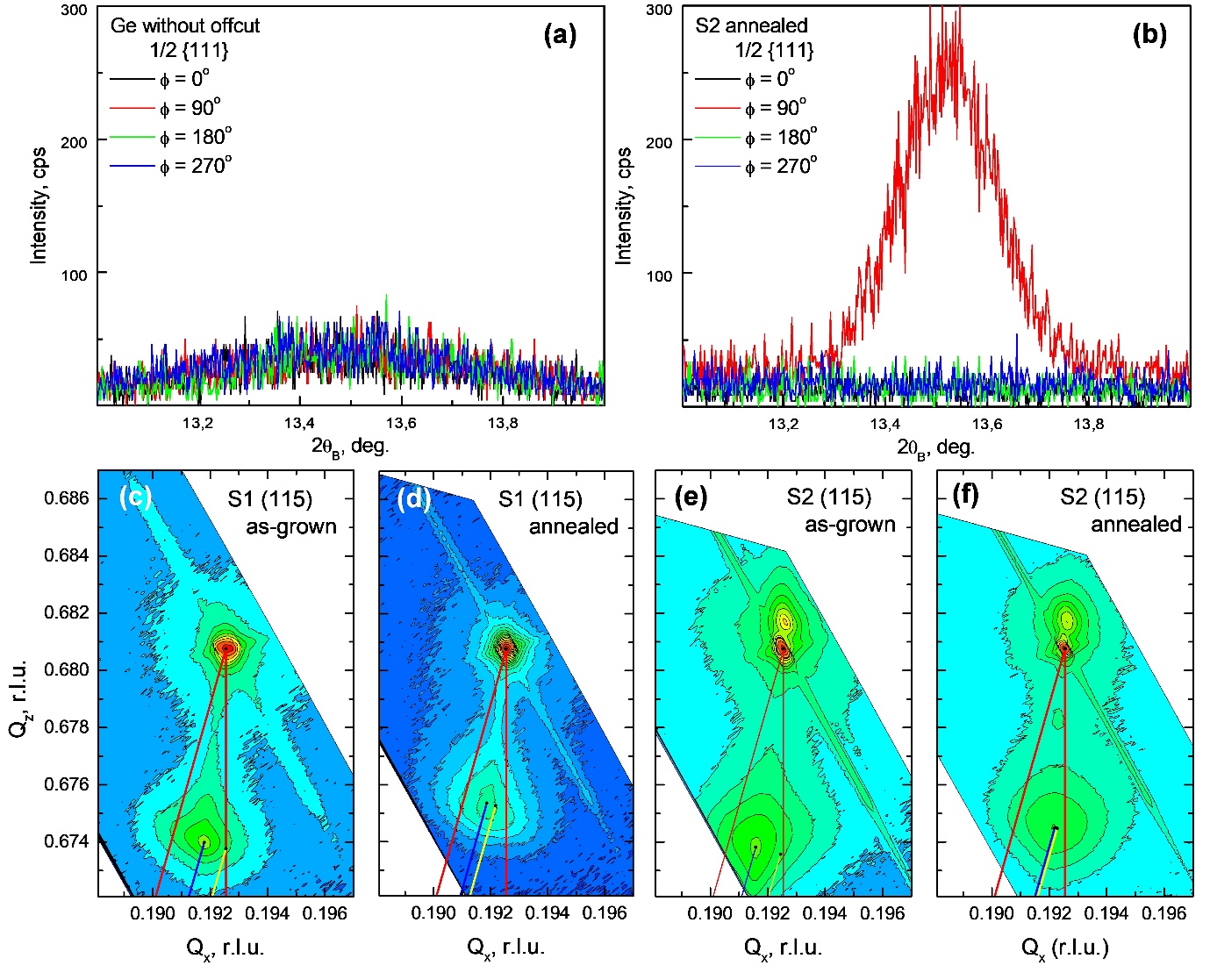

Supplement: Supplementary file 1 — Supplementary information. [file 41598_2020_58812_MOESM1_ESM.docx]
